# Exploring the Connection Between Substance Use and Mental Health in Brazilian Teens Who Have Experienced Sexual Violence

**DOI:** 10.1002/ijop.70133

**Published:** 2025-11-03

**Authors:** Adriana Scatena, Lucas da Rosa Ferro, Laura Soares da Silva, José Eugenio Rodríguez Fernández, Wanderlei Abadio de Oliveira, André Luiz Monezi Andrade

**Affiliations:** ^1^ Psychology Section, Santo André Medical Center Santo Andre Brazil; ^2^ Department of Psychobiology Universidade Federal de São Paulo São Paulo Brazil; ^3^ School of Life Sciences Pontifical Catholic University of Campinas Campinas Brazil; ^4^ Facultad de Ciencias de la Educación Universidad de Santiago de Compostela Santiago de Compostela Spain

**Keywords:** adolescents, mental health, sexual violence, substance use, victimisation

## Abstract

A large study involving 129,953 adolescents (aged 13–17) examined the connection between sexual violence, substance use, and mental health. Victims of sexual violence (VSV; *n* = 20,492; 14.8%) and rape (RV; *n* = 8133; 6.5%) reported higher rates of parental or caregiver alcohol and cigarette use. These adolescents were also more likely to engage in substance use themselves, especially alcohol and illicit drugs, often beginning before age 14. They also faced increased exposure to risky behaviours like drinking with classmates and using illegal drugs. Additionally, the VSV and RV groups reported significantly higher levels of sadness, anger, suicidal thoughts, and poorer overall health. This research, based on data from a large‐scale survey conducted in Brazil, underscores the urgent need for targeted interventions and support systems to address the complex challenges faced by adolescents who have experienced sexual violence.

## 1 Introduction

Sexual violence victimisation (SVV) is a serious violation of human rights (Hayes and Maher 2023) and remains a widespread and deeply rooted societal problem. SVV is naturally complex and includes various behaviours (Miele et al. 2016). Among these are sexual abuse and rape, with the former involving unwarranted sexual advances, remarks, or insinuations of an offensive nature that create a hostile environment for the victim (Mathews and Collin‐Vézina 2019), and the latter defined by non‐consensual invasive penetration (World Health Organization 2013).

Within the scientific literature, it is well established that SVV disproportionately affects women (Cepeda et al. 2022). This higher prevalence among females can be linked to several factors, including gender inequality (LeSuer 2022), the persistence of culturally accepted behaviours and norms (Başak and Bulut Serin 2023), and socio‐economic factors combined with weaknesses in legislative and public policy frameworks (Rockowitz et al. 2023). Importantly, besides women, young people and adolescents are especially vulnerable to SVV. Alarming statistics highlight this vulnerability. Specifically, the United Nations Children's Fund report states that 9.6% of the 204 million children under 18 experience sexual exploitation, 22.9% suffer from physical abuse, and 29.1% face emotional distress (UNICEF 2020). The report also emphasises the deeply rooted societal taboos surrounding SVV in many cultures, which contribute to widespread underreporting and hinder the development of effective local laws.

Of importance to the present study is a substantial body of research that has identified the link between SVV and indicators of poor mental health (Rollison et al. 2023; Wang et al. 2023). An integrative review conducted by Campbell et al. (2009) highlighted the significant mental health distress experienced by SVV survivors, notably post‐traumatic stress disorder (PTSD), which has a prevalence range of 17% to 65%. Depression and anxiety are also common, with rates ranging from 13% to 51% for depression and 12% to 40% for anxiety. The review also revealed that substance use issues are prevalent among SVV survivors, with alcohol use disorders found in 13%–49% of cases and drug use disorders in 28%–61%. Alarmingly, suicidality is also frequent, with 23%–44% of survivors reporting suicidal ideations and 2%–19% attempting suicide. A more recent meta‐analysis by Dworkin et al. (2017) confirmed these findings, showing a strong relationship between SVV and various forms of mental health distress, with suicidality being the most prevalent.

Nevertheless, the connection between SVV and problematic substance use (PSU) remains a subject of ongoing research (Phelan 2023; Mintz et al. 2022; Quigg et al. 2020; Cammack et al. 2019). Notably, alcohol has received significant attention in studies of SVV (Siconolfi et al. 2023; Badour et al. 2020; Duval et al. 2020). Some researchers suggest that, because of alcohol's depressant effects on the nervous system, it may serve as a form of self‐medication to reduce emotional distress and trauma caused by SVV (Hawn et al. 2020). However, consensus on this issue remains unclear in the literature. For example, a prospective study by Testa et al. (2007) examined whether SVV was linked to increased alcohol use among women. The results showed that SVV survivors did not have higher alcohol consumption after their victimisation. Moreover, among women experiencing SVV, those with PTSD did not consume more alcohol than those without PTSD. Additionally, there may be a two‐way relationship between substance use and SVV, such that certain social settings associated with substance use (e.g., nightclubs) might increase the risk of SVV incidents (Baldwin et al. 2022; Miller et al. 2022; Quigg et al. 2020; Anda et al. 1999).

A notable gap in the current research on the connection between SVV, PSU, and mental health distress is the scarcity of epidemiological studies with representative samples. However, one such study by Xu et al. (2013) identified female gender, solitary living, economic hardship, childhood adversity, and parental psychopathology as risk factors for SVV. The study also indicated that SVV survivors are more vulnerable to PTSD and PSU. Although valuable, Xu et al. (2013) only examined alcohol and nicotine, and the sample included only adults.

Overall, there is a gap in the research on the relationship between SVV, PSU, and mental health issues among adolescents using representative samples. In the context of Brazil, it is notable that SVV was only evaluated in the second‐to‐last round of the National School Health Survey (Pesquisa Nacional de Saúde do Escolar or PeNSE), conducted by the Brazilian Institute of Geography and Statistics (IBGE 2016) in 2016. The PeNSE is an epidemiological study that employs probabilistic sampling to understand the experiences of Brazilian adolescents aged 13–17. The most recent version of the PeNSE survey was carried out in 2022 with a large sample size of nearly 160,000 adolescents. Additionally, the operational definition of SVV was expanded to include cases of rape victimization alongside sexual abuse (IBGE 2022).

The findings from this recent PeNSE edition showed that 14.6% of adolescents experienced some form of sexual abuse at least once in their lifetime. Notably, this rate was nearly twice as high in females (20.1%) compared to males (9.0%). Additionally, the overall prevalence of rape victimisation was 6.3%, with a significantly higher occurrence among girls (8.8%) than boys (3.6%). Similarly, regarding emotional distress, the report indicated that negative self‐perceptions of mental health were nearly three times more common among girls (27%) than boys (8%). Furthermore, girls were more likely to report feelings of life being meaningless (29.6%) compared to boys (13%). In terms of substance use, there was a concerning rise in alcohol and illicit drug use, increasing by over 10% and 4%, respectively, from 2012 to 2019, with the largest increase observed among girls. Moreover, the prevalence of adolescents engaging in substance use before age 14 almost doubled from 2009 (3.4%) to 2019 (5.4%). However, to our knowledge, no study has yet examined the relationship between SVV, PSU, and mental health using PeNSE data.

The present study aimed to address this gap by examining possible connections among Brazilian adolescents who have experienced SVV, particularly those who have reported at least one incident of sexual abuse or rape, and their involvement in substance use as well as the appearance of PMHI indicators.

## 2 Method

### 2.1 Participants and Procedure

The current study involved a secondary data analysis using the fourth edition of the National School Health Survey (PeNSE), conducted by the Brazilian Institute of Geography and Statistics (IBGE) in 2022. Although the survey started in 2019, access to the microdata was only granted in late 2022. This delay was necessary to ensure compliance with the ‘General Data Protection Law,’ enacted in Brazil in 2020, which coincided with the typical release of the microdata. Adolescents were sampled from 4253 schools, both public and private, through a probabilistic sampling method to represent the broader population of Brazilian adolescents aged 13–17. While the survey initially involved 159,000 respondents, this study concentrated on the 129,953 adolescents who answered all items on the questionnaire fully.

### 2.2 Measures

PeNSE covers a wide range of areas for adolescent assessment, including psychosocial aspects like drug use (both legal and illegal), mental health, the quality of school and family environments, physical activity, body image and patterns of healthcare service use. A key feature of PeNSE is its ability to compare data internationally, made possible through alignment with the Global School‐Based Student Health Survey (GSHS) conducted by the World Health Organization (WHO) and the Centers for Disease Control and Prevention (CDC), which are carried out in about 90 countries.

For the current study, adolescents who answered ‘yes’ to the question ‘Have you ever been touched, manipulated, kissed, or had body parts exposed against your will?’ were classified as victims of sexual violence (VSV; *n* = 20,492; 14.8% of the total sample). Those who responded negatively were categorised as non‐victims of sexual violence (nVSV; *n* = 109,461; 85.2% of the total sample). Regarding rape, adolescents who responded ‘yes’ to the question ‘Have you ever been threatened, intimidated, or coerced into engaging in sexual intercourse or any other sexual act against your will?’ were classified as victims of rape (RV; *n* = 8,133; 6.5% of the total sample), while those who responded negatively were categorised as non‐victims of rape (nRV; *n* = 121,820; 93.5% of the total sample).

### 2.3 Data Analysis

Counts were considered first in descriptive statistics, along with percentages and confidence intervals based on the weighted sample. To account for the complex survey design, we used the Rao–Scott Chi‐Square test to analyze categorical variables. We also performed additional analyses using both crude and adjusted logistic regressions, as shown in Table S1. The adjusted odds ratios were controlled for sex, age, school location (urban or rural), and school type (private or public).

A *t*‐test was conducted on continuous variables using independent samples. Cohen's D was utilised to measure observed differences. To promote transparency and reproducibility, we have shared the following files via the Open Science Framework (OSF): (1) A script listing all analysed variables; (2) Two Excel files in the OSF repository containing all research results. These spreadsheets include additional information that could not be included in the tables due to space limitations; (3) A template of all tables in the manuscript with corresponding codes for each variable, aiding readers in understanding and comparing codes; (4) The complete study database.

All data are available at the following address: https://osf.io/rzm7a/?view_only=8812219829b0432e8d45732b32b0c926.

All statistical analyses were conducted using R and SAS software, with the ‘Survey’ package, as recommended in the IBGE report (2022). This specialised package is designed for analysing probabilistic sampling data. It incorporates sampling weights to enhance the generalizability of the results to the broader population of Brazilian adolescents.

## 3 Results

Regarding substance use (Table 1), individuals in the VSV group showed a significantly higher rate of parental and/or caregiver alcohol consumption (either one or both) compared to their nVSV counterparts. This pattern also applies to cigarette use among parents or caregivers. Furthermore, when looking at lifetime substance use, the VSV group demonstrated a significantly higher prevalence of substance use, including alcohol and illicit drugs. Additionally, within the VSV group, early exposure to cigarettes, alcohol, and illicit substances before age 14 was significantly more common than in the nVSV group. Similarly, the VSV group's rate of engaging in risky behaviours in the past 30 days was also higher. Nearly 60% of adolescents in the VSV group reported witnessing a classmate drink alcohol, while 32% reported encountering illicit drugs nearby.

**TABLE 1:** Differences in substance use between adolescents identified as victims of sexual violence (VSV, *n* = 20,492) and those not involved in sexual violence (nVSV, *n* = 109,461).

	Groups									
	VSV					nVSV				
		Weighted					Weighted			
	Overall unweighted counts	%	LL 95% CI	UL 95% CI		Overall unweighted counts	%	LL 95% CI	UL 95% CI	*p*
Lifetime										
Alcohol										***
Yes	16,314	81.2	80.0	82.3		66,098	62.4	61.8	63.0	
No	4178	18.8	17.7	20.0		43,363	37.6	37.0	38.2	
Cigarettes										***
Yes	7029	38.3	36.9	39.8		20,784	21.6	21.1	22.1	
No	13,463	61.7	60.2	63.1		88,677	78.4	77.9	78.9	
Illicit drugs										***
Yes	4766	25.7	24.4	27.1		11,818	12.1	11.7	12.5	
No	15,726	74.3	72.9	75.6		97,643	87.9	87.5	88.3	
Past month										
Has any friend drunk in your presence?										***
Yes	11,467	59.4	58.0	60.9		43,599	42.7	42.1	43.4	
No	9025	40.6	39.1	42.0		65,862	57.3	56.6	57.9	
Has any friend used illicit drugs in your presence?										***
Yes	5661	31.7	30.3	33.2		15,481	15.9	15.4	16.3	
No	14,831	68.3	66.8	69.7		93,980	84.1	83.7	84.6	

	Overall unweighted counts	Mean	LL 95% CI	UL 95% CI		Overall unweighted counts	Mean	LL 95% CI	UL 95% CI	*p*
Onset										
Cigarette	7012	13.41	13.36	13.46		20,705	13.63	13.60	13.66	***
Alcohol	16,289	12.88	12.85	12.91		66,001	13.18	13.16	13.19	***
Illicit substances	4744	14.14	14.09	14.20		11,754	14.41	14.38	14.45	***
Frequency of alcohol‐related disruptions in relationships, classes, or conflicts	16,310	0.83	0.79	0.86		66,066	0.45	0.43	0.46	***
Days used in the past days										
Cigarette	7017	2.56	2.40	2.72		20,745	2.11	2.03	2.20	***
Alcohol	16,303	2.45	2.38	2.53		66,055	1.78	1.75	1.81	***
Any drugs	4761	1.60	1.52	1.69		11,802	1.51	1.46	1.57	***
Cannabis	4758	1.25	1.19	1.31		11,801	1.17	1.14	1.21	***
Crack/cocaine	4756	0.21	0.18	0.24		11,791	0.08	0.06	0.09	***
Past month										
Number of alcoholic drinks or daily alcohol doses	8100	3.02	2.99	3.06		27,051	2.83	2.81	2.85	***

*Note:* % represents the proportion indicative of the population. The *p*‐value indicates the level of statistical significance for the Rao–Scott Chi‐Square Test, which is the design‐adjusted equivalent of the Pearson Chi‐square test.

Abbreviations: LL 95% CI: lower confidence interval; UL 95% CI: upper confidence interval.

*** The *p*‐value is less than 0.001, indicating a highly significant result.

The pattern of results concerning substance use remained consistent when comparing victims of rape (RV) and non‐victims of rape (nRV) (see Table 2). Specifically, adolescents who were victims of rape reported a higher prevalence of parental alcohol and cigarette use. Similarly, the RV group showed a significantly higher rate of substance use, including alcohol, cigarettes, vaping, cannabis, and other illegal drugs. Early exposure to substance use was also notably more common in the RV group, as was exposure to risky behaviours such as using drugs with friends and participating in situations with individuals under the influence of alcohol.

**TABLE 2:** Differences in substance use between adolescents categorised as rape victims (RV, *n* = 8133) and non‐rape victims (nRV, *n* = 121,820).

	Groups									
	RV					nRV				
	Overall unweighted counts	Weighted				Overall unweighted counts	Weighted			
		%	LL 95% CI	UL 95% CI			%	LL 95% CI	UL 95% CI	*p*
Lifetime										
Alcohol										***
Yes	6667	83.2	81.6	84.8		75,745	63.9	63.3	64.5	
No	1466	16.8	15.2	18.4		46,075	36.1	35.5	36.7	
Cigarettes										***
Yes	3447	45.9	43.7	48.2		24,366	22.6	22.1	23.1	
No	4686	54.1	51.8	56.3		97,454	77.4	76.9	77.9	
Illicit drugs										***
Yes	2414	32.8	30.5	35.0		14,170	12.8	12.4	13.3	
No	5719	67.2	65.0	69.5		107,650	87.2	86.7	87.6	
Past month										
Has any friend drunk in your presence?										***
Yes	4890	63.8	61.6	65.9		50,176	43.9	43.3	44.5	
No	3243	36.2	34.1	38.4		71,644	56.1	55.5	56.7	
Has any friend used illicit drugs in your presence?										***
Yes	2750	36.4	34.1	38.6		18,392	17.0	16.5	17.4	
No	5383	63.6	61.4	65.9		103,428	83.0	82.6	83.5	

	Overall unweighted counts	Mean	LL 95% CI	UL 95% CI		Overall unweighted counts	Mean	LL 95% CI	UL 95% CI	*p*
Onset										
Cigarette	3436	13.33	13.26	13.40		24,281	13.60	13.58	13.63	***
Alcohol	6662	12.84	12.78	12.89		75,628	13.14	13.13	13.16	***
Illicit substances	2399	13.92	13.84	14.00		14,099	14.41	14.38	14.44	***
Frequency of alcohol‐related disruptions in relationships, classes, or conflicts.	6665	1.06	1.00	1.12		75,711	0.47	0.46	0.49	***
Days used in the past days										
Cigarette	3443	3.51	3.24	3.78		24,319	2.04	1.96	2.12	***
Alcohol	6658	3.15	3.00	3.29		75,700	1.81	1.78	1.84	***
Any drugs	2411	1.91	1.78	2.04		14,152	1.48	1.43	1.52	***
Cannabis	2410	1.47	1.38	1.57		14,149	1.15	1.11	1.18	***
Crack/Cocaine	2408	0.35	0.30	0.41		14,139	0.07	0.06	0.08	***
Past month										
Number of alcoholic drinks or daily alcohol doses	3543	3.13	3.08	3.19		31,608	2.85	2.83	2.87	***

*Note:* % represents the proportion representative of the population. *p*‐value signifies the level of statistical significance for the Rao–Scott Chi‐Square Test, which is the design‐adjusted equivalent of the Pearson Chi‐square test.

Abbreviations: LL 95% CI: lower level confidence interval; UL 95% upper level confidence interval.

*** The *p*‐value is less than 0.001, indicating a highly significant result.

Turning to mental health indicators (Table 3), nearly 40% of participants in the VSV group reported that their parents rarely or never have access to their school routines or academic activities. Additionally, almost 30% of the VSV group believed that their parents understood their concerns and problems, while this figure was nearly double (around 50%) in the nVSV group. Moreover, the VSV group showed nearly twice the proportion of individuals who felt that nobody cared about them. Feelings of sadness, anger, suicidal thoughts, and a poorer perception of overall health were also significantly more common in the VSV group.

**TABLE 3:** Differences in emotional distress between adolescents categorised as victims of sexual violence (VSV, *n* = 20,492) and non‐victims of sexual violence (nVSV, *n* = 109,461).

	Groups									
	VSV					nVSV				
		Weighted					Weighted			
	Overall unweighted counts	%	LL 95% CI	UL 95% CI		Overall unweighted counts	%	LL 95% CI	UL 95% CI	*p*
Past month										
How often did your parent or guardian comprehend your concerns?										***
Rarely	9546	47.6	46.1	49.1		33,300	30.1	29.6	30.7	
Sometimes	4603	22.1	20.9	23.4		24,148	21.4	20.9	21.9	
Most of the time	3809	17.2	16.1	18.3		26,552	22.7	22.2	23.2	
Always	2534	13.1	12.1	14.1		25,461	25.8	25.2	26.3	
How often did you feel sad?										***
Rarely	3385	17.3	16.2	18.4		39,137	36.8	36.2	37.4	
Sometimes	6634	30.7	29.3	32.0		39,636	34.9	34.3	35.5	
Most of the time	6058	28.4	27.0	29.7		20,380	18.3	17.8	18.8	
Always	4415	23.6	22.4	24.9		10,308	9.9	9.5	10.3	
How often did you feel that no one cared about you?										***
Rarely	5987	28.9	27.5	30.2		53,858	48.4	47.7	49.0	
Sometimes	5397	24.1	22.9	25.4		26,909	24.3	23.8	24.8	
Most of the time	4758	23.8	22.5	25.0		16,382	15.1	14.6	15.5	
Always	4350	23.2	22.0	24.5		12,312	12.2	11.8	12.7	
How often did you experience irritation, anger, or a negative mood?										***
Rarely	2980	15.3	14.3	16.3		28,707	27.8	27.2	28.4	
Sometimes	5689	26.2	24.9	27.5		38,274	33.7	33.1	34.3	
Most of the time	6241	29.0	27.7	30.3		25,839	22.6	22.1	23.1	
Always	5582	29.5	28.1	30.9		16,641	15.9	15.4	16.4	
How often did you sense life's lack of value?										
Rarely	9042	42.1	40.7	43.6		73,683	65.7	65.1	66.3	
Sometimes	4174	20.1	18.9	21.3		16,824	15.7	15.3	16.2	
Most of the time	3389	17.2	16.1	18.4		9821	9.2	8.9	9.6	
Always	3887	20.6	19.4	21.8		9133	9.4	9.0	9.7	
How would you rate your health?										***
Very good	4172	22.5	21.2	23.7		34,178	33.6	33.0	34.2	
Good	6783	31.6	30.2	33.0		42,455	37.9	37.3	38.6	
Fair	7125	34.8	33.4	36.2		27,577	24.2	23.7	24.7	
Bad	1794	8.4	7.6	9.2		4044	3.3	3.0	3.5	
Very bad	618	2.8	2.3	3.2		1207	1.0	0.9	1.1	

*Note:* % indicates the proportion representing the population. *p*‐value indicates the level of statistical significance for the Rao–Scott Chi‐Square Test, which is the design‐adjusted version of the Pearson Chi‐square test.

Abbreviations: LL 95% CI: lower limit of the confidence interval; UL 95% CI: upper limit of the confidence interval.

*** The *p*‐value is less than 0.001, showing a highly significant result.

Similar patterns were detected among adolescents in the RV group (see Table 4). Just over 50% of participants in this group reported that their parents rarely or never knew about their school routines or academic activities. In contrast, about 30% of the nRV group reported the same. Additionally, within the RV group, there was a higher occurrence of sadness, anger, suicidal thoughts, and a more negative view of health.

**TABLE 4:** Differences in emotional distress between adolescents categorised as rape victims (RV, *n* = 8133) and non‐rape victims (nRV, *n* = 121,820).

	Groups									
	RV					nRV				
		Weighted					Weighted			
	Overall unweighted counts	%	LL 95% CI	UL 95% CI		Overall unweighted counts	%	LL 95% CI	UL 95% CI	*p*
Past month										
How often did your parent or guardian comprehend your concerns?										***
Rarely	4227	51.9	49.7	54.2		38,619	31.4	30.8	32.0	
Sometimes	1639	20.3	18.4	22.1		27,112	21.6	21.1	22.1	
Most of the time	1234	14.2	12.6	15.8		29,127	22.4	21.9	22.9	
Always	1033	13.6	12.0	15.2		26,962	24.6	24.1	25.1	
How often did you feel sad?										***
Rarely	1212	16.6	14.9	18.3		41,310	35.2	34.6	35.7	
Sometimes	2179	25.0	23.1	26.9		44,091	35.0	34.4	35.5	
Most of the time	2482	30.1	28.0	32.3		23,956	19.1	18.6	19.6	
Always	2260	28.3	26.3	30.4		12,463	10.8	10.4	11.2	
How often did you feel that no one cared about you?										***
Rarely	2082	25.1	23.2	27.0		57,763	46.9	46.3	47.5	
Sometimes	1924	21.9	20.1	23.8		30,382	24.4	23.9	25.0	
Most of the time	1960	24.6	22.6	26.6		19,180	15.8	15.3	16.2	
Always	2167	28.4	26.3	30.5		14,495	12.9	12.5	13.3	
How often did you experience irritation, anger, or a negative mood?										***
Rarely	1309	17.2	15.5	18.8		30,378	26.6	26.0	27.1	
Sometimes	1960	23.6	21.7	25.5		42,003	33.2	32.7	33.8	
Most of the time	2424	28.4	26.3	30.4		29,656	23.2	22.7	23.7	
Always	2440	30.9	28.7	33.0		19,783	17.0	16.5	17.5	
How often did you sense life's lack of value?										
Rarely	2917	35.0	32.8	37.1		79,808	64.1	63.5	64.7	
Sometimes	1601	18.2	16.5	19.9		19,397	16.2	15.8	16.7	
Most of the time	1503	19.2	17.4	21.0		11,707	9.8	9.4	10.2	
Always	2112	27.6	25.5	29.7		10,908	9.9	9.5	10.2	
How would you rate your health?										***
Very good	1617	23.1	21.2	25.1		36,733	32.6	32.0	33.1	
Good	2404	28.7	26.7	30.8		46,834	37.6	37.0	38.2	
Fair	2874	34.0	31.8	36.1		31,828	25.2	24.7	25.7	
Bad	846	9.3	8.1	10.6		4992	3.7	3.4	3.9	
Very bad	392	4.8	3.9	5.7		1433	1.0	0.9	1.1	

*Note:* % represents the proportion representative of the population. *p*‐value signifies the level of statistical significance for the Rao–Scott Chi‐Square test, which is the design‐adjusted equivalent of the Pearson Chi‐square test.

Abbreviations: LL 95% CI: lower level confidence interval; UL 95% upper level confidence interval.

*** The *p*‐value is less than 0.001, indicating a highly significant result.

## 4 Discussion

This study investigated the links between SVV, substance use, and mental health in a representative sample of Brazilian adolescents. Adolescents in both the VSV and RV groups showed similar rates of drug use and mental distress, which were significantly higher than those in the non‐VSV and non‐RV groups, consistent with prior research (Rollison et al. 2023; Wang et al. 2023). However, in this study, we identified specific substance use patterns among adolescents who have experienced sexual violence (Mintz et al. 2022). Notably, a higher rate of alcohol consumption was found among parents in the VSV and RV groups. Several studies have reported an increased risk of SVV when perpetrators consume alcohol (Bourey et al. 2023; Ingram et al. 2022).

The increased rates of alcohol consumption among adolescents in the VSV and RV groups, along with their reports of friends drinking in their presence, highlight the potential risk for sexual assaults, especially in social settings like nightclubs (Baldwin et al. 2022). A systematic review of 69 articles examining SVV in nightlife settings found that both alcohol and illicit drugs were among the strongest factors linked to SVV (Quigg et al. 2020). These findings are important, particularly as a much higher prevalence of illicit drug use was also observed among adolescents in the VSV and RV groups. Additionally, teens in these groups smoked and used hookah more often. While the PeNSE survey did not explore the context of drug use among adolescents (such as at parties, in nightlife venues, or barbecues), it's important to recognise that exposure to these substances can significantly increase the risk of SVV. This is because potential perpetrators of sexual assault may be present in the same environment as victims (Miller et al. 2022). This risk may be especially high in places where people gather to smoke, like outdoor areas of bars and clubs (Anda et al. 1999).

There is also evidence to suggest that childhood SVV may increase the likelihood of smoking in adulthood (Kristman‐Valente et al. 2013) and that parents' or caregivers' smoking behaviour may be linked to sexual violence (Cammack et al. 2019; Mintz et al. 2022). However, it is important to recognise that the causality between SVV and smoking behaviour cannot be definitively established, as PeNSE is a cross‐sectional study. Additionally, many adolescents who experienced SVV reported multiple perpetrators (including parents, boyfriends, friends, etc.), and the PeNSE survey only assessed cigarette smoking among the adolescents and their parents, which excludes other potential perpetrators of sexual violence.

A cohort study involving over 67,000 American nurses revealed a strong link between sexual abuse and adult mortality, especially among those who smoked or had higher depression levels (Wang et al. 2023). Consistent with existing research (Rollison et al. 2023; Campbell et al. 2009), our study also found a strong connection between SVV and mental health issues, such as PTSD. One of the most important outcomes of SVV is its association with post‐traumatic stress symptoms (Hawn et al. 2020; Testa et al. 2007), which can lead to fear and helplessness (Xu et al. 2013). These symptoms may last long‐term and contribute to anxiety and depression disorders. Anxiety is also a common response to SVV. Victims frequently feel constantly threatened and expect imminent dangers, which impact their quality of life and their ability to function normally (Dworkin et al. 2017). Depressive disorders can cause intense sadness, hopelessness, a loss of interest in daily activities, and social withdrawal.

SVV represents a traumatic experience with far‐reaching repercussions, significantly impacting various aspects of victims' lives. The most profound effects are felt within interpersonal relationships. SVV survivors often face substantial challenges in establishing and maintaining healthy, fulfilling relationships. These traumatic experiences can cause difficulties in trust, intimacy, and communication, hindering their ability to form meaningful connections with others (Ten Have et al. 2019). As a result, this can lead to social isolation and alienation.

In social functioning, failure to develop practical social skills after SVV can lead to long‐term isolation and difficulty forming meaningful bonds with others (Mereish and Poteat 2015). Substance use, such as alcohol and cigarettes, might help facilitate social interaction, temporarily relieving social anxiety and creating a sense of belonging (Phelan 2023). Therefore, the combination of societal isolation and social skill deficiencies can significantly increase the risk of drug use among individuals who have experienced SVV.

It is important to recognise the limitations of this study when interpreting its findings. Primarily, this study uses a cross‐sectional design. This limitation prevents the determination of causal links between SVV, substance use, and mental health issues. Although significant associations are found, it is unclear whether SVV directly leads to substance use or mental health problems, or if these factors influence the occurrence of SVV. Therefore, the conclusions should be drawn carefully, and future longitudinal studies may help clarify the complex relationships among these variables.

## 5 Conclusions

In this study, SVV adolescents showed significantly higher rates of alcohol, tobacco, and illicit drug use, including early initiation and increased exposure to risky environments. It was also noted that both the VSV and RV groups experienced more feelings of sadness, anger, suicidal thoughts, and negative views of their own health compared to non‐victims. Regarding future research, they could examine how psychosocial variables related to SVV and SVV have changed over time by comparing different editions of PeNSE to see if the connection between sexual violence and risk behaviours in adolescents has become stronger in recent years. Although this is a quantitative study, it is also recommended to explore these relationships more thoroughly through qualitative methods, which can complement the quantitative results by offering insights into adolescents' experiences and perspectives. This approach can help clarify the psychosocial mechanisms behind the strong link observed between sexual violence, substance use, and psychological distress.

## Author Contributions

A.S., L.R.F., and A.L.M.A. were responsible for the study design. L.R.F., A.S. and L.S.S. were responsible for data analyses. A.S., J.E.R.F., W.A.O. and A.L.M.A. cooperated in the technical procedures, interpretation of the data, and preparation of the manuscript. All the authors are responsible for their contents, having revised and approved its final version.

## Ethics Statement

This study's methodology, which utilised a public secondary database, did not require additional approval from our institution's Research Ethics Committee. This exemption is based on prior approval granted under protocol number 3,249,268, dated August 4, 2019, by a relevant ethics oversight body. This approval aligns with Article 1, item II of Resolution 560/2016 by the National Health, which allows research using publicly available or secondary data as defined in Law No. 12.527 of November 18, 2011. The original PeNSE 2019 survey was approved by the Brazilian National Commission on Ethics in Research (CONEP), as documented by the Brazilian Institute of Geography and Statistics (IBGE), and was conducted in accordance with the Statute of the Child and Adolescent (ECA). Thus, using the PeNSE data within the IBGE database fully complies with ethical standards and legal requirements, negating the need for further ethical review. Additionally, this approval followed the ethical principles of the Declaration of Helsinki (1964) and its subsequent amendments, ensuring respect for human dignity, privacy, and participant confidentiality.

## Consent

Informed consent was obtained from all individual adult participants included in the study by IBGE.

## Conflicts of Interest

The authors declare no conflicts of interest.

## Supporting information


**Table S1:** Logistic regression models considering both adjusted and crude odds ratios for substance use among adolescents categorised as victims of sexual violence (VSV, *n* = 20,492) and non‐victims of sexual violence (nVSV, *n* = 109,461) (Variable code: 160E). *Note:* Model adjusted for gender, age, school location (urban or rural), and type of school (public or private). REF = reference value. *** *p* < 0.001.
**Table S2:** Logistic regression models considering the adjusted and crude odds ratios in substance use between adolescents categorised as rape victims (RV, *n* = 8133) and non‐rape victims (nRV, *n* = 121,820). (Variable code: 167E). *Note:* Model adjusted for gender, age, school location (urban or rural), and type of school (public or private). REF = reference value. *** *p* < 0.001.
**Table S3:** Logistic regression models considering adjusted and crude odds ratios for emotional distress between adolescents categorised as victims of sexual violence (VSV, *n* = 20,492) and non‐victims of sexual violence (nVSV, *n* = 109,461). (Variable code: 160E). *Note:* Model adjusted for gender, age, school location (urban or rural), and type of school (public or private). REF = reference value. *** *p* < 0.001.
**Table S4:** Logistic regression models examining the adjusted and crude odds ratios for emotional distress between adolescents categorised as rape victims (RV, *n* = 8133) and non‐rape victims (nRV, *n* = 121,820). (Variable code: 167E). *Note:* Model adjusted for gender, age, school location (urban or rural), and type of school (public or private). REF = reference value. * *p <* 0.05; *** *p <* 0.001.

## Data Availability

The data generated and analyzed in this study are accessible through the Open Science Framework at the provided link: https://osf.io/rzm7a/?view_only=8812219829b0432e8d45732b32b0c926.
