# Supplementary material for: Exploring the Connection Between Substance Use and Mental Health in Brazilian Teens Who Have Experienced Sexual Violence
Source: Int J Psychol. 2025 Nov 3;60(6):e70133. doi: 10.1002/ijop.70133 (PMC12583886; doi:10.1002/ijop.70133)
Supplement: Supplementary file 1 — Table S1: Logistic regression models considering both adjusted and crude odds ratios for substance use among adolescents categorised as victims of sexual violence (VSV, n = 20,492) and non‐victims of sexual violence (nVSV, n = 109,461) (Variable code: 160E). Note: Model adjusted for gender, age, school location (urban or rural), and type of school (public or private). REF = reference value. *** p < 0.001. Table S2: Logistic regression models considering the adjusted and crude odds ratios in substance use between adolescents categorised as rape victims (RV, n = 8133) and non‐rape victims (nRV, n = 121,820). (Variable code: 167E). Note: Model adjusted for gender, age, school location (urban or rural), and type of school (public or private). REF = reference value. *** p < 0.001. Table S3: Logistic regression models considering adjusted and crude odds ratios for emotional distress between adolescents categorised as victims of sexual violence (VSV, n = 20,492) and non‐victims of sexual violence (nVSV, n = 109,461). (Variable code: 160E). Note: Model adjusted for gender, age, school location (urban or rural), and type of school (public or private). REF = reference value. *** p < 0.001. Table S4: Logistic regression models examining the adjusted and crude odds ratios for emotional distress between adolescents categorised as rape victims (RV, n = 8133) and non‐rape victims (nRV, n = 121,820). (Variable code: 167E). Note: Model adjusted for gender, age, school location (urban or rural), and type of school (public or private). REF = reference value. * p < 0.05; *** p < 0.001. [file IJOP-60-e70133-s001.docx]

**SUPPLEMENTARY TABLES**

**Supplementary Table 1**

Logistic regression models considering the adjusted and crude odds ratio in substance use among adolescents categorized as victims of sexual violence (VSV, n=20,492) and non-victims of sexual violence (nVSV, n=109,461) (Variable code: 160E).

| **VARIABLES CODED** |  | **Adjusted OR** | | | |  | **Crude OR** | | | |
| --- | --- | --- | --- | --- | --- | --- | --- | --- | --- | --- |
|  |  |  | **95% CI** | |  |  |  | **95% CI** | |  |
| **LIFETIME** |  |  | **Lower** | **Upper** | ***p*** |  |  | **Lower** | **Upper** | ***p*** |
| Alcohol (V085) |  |  |  |  | *** |  |  |  |  | *** |
| Yes |  | 2.41 | 2.21 | 2.63 |  |  | 1.71 | 1.55 | 1.89 |  |
| No |  | REF |  |  |  |  | REF |  |  |  |
| Cigarettes (V067) |  |  |  |  | *** |  |  |  |  | ******* |
| Yes |  | 2.32 | 2.14 | 2.52 |  |  | 1.34 | 1.21 | 1.49 |  |
| No |  | REF |  |  |  |  | REF |  |  |  |
| Illicit drugs (V094) |  |  |  |  | *** |  |  |  |  | ******* |
| Yes |  | 2.63 | 2.38 | 2.90 |  |  | 1.36 | 1.20 | 1.54 |  |
| No |  | REF |  |  |  |  | REF |  |  |  |
| **PAST MONTH** |  |  |  |  |  |  |  |  |  |  |
| Has any friend drunk in your presence? (V093) |  |  |  |  | *** |  |  |  |  | ******* |
| Yes |  | 1.94 | 1.80 | 2.09 |  |  | 1.28 | 1.18 | 1.39 |  |
| No |  | REF |  |  |  |  | REF |  |  |  |
| Has any friend used illicit drugs in your presence? (V099) |  |  |  |  | *** |  |  |  |  | ******* |
| Yes |  | 2.61 | 2.39 | 2.85 |  |  | 1.63 | 1.47 | 1.81 |  |
| No |  | REF |  |  |  |  | REF |  |  |  |

*Note:* Model adjusted for gender, age, school location (urban or rural) and type of school (public or private). REF = reference value. *** p<0.001.

**Supplementary Table 2**

Logistic regression models considering the adjusted and crude odds ratio in substance use between adolescents categorized as rape victims (RV, n= 8,133) and non- rape victims (nRV, n= 121,820).

(Variable code: 167E).

| **VARIABLES CODED** |  | **Adjusted OR** | | | |  | **Crude OR** | | | |
| --- | --- | --- | --- | --- | --- | --- | --- | --- | --- | --- |
|  |  |  | **95% CI** | |  |  |  | **95% CI** | |  |
| **LIFETIME** |  |  | **Lower** | **Upper** | ***p*** |  |  | **Lower** | **Upper** | ***p*** |
| Alcohol (V085) |  |  |  |  | *** |  |  |  |  | *** |
| Yes |  | 2.55 | 2.24 | 2.91 |  |  | 1.57 | 1.35 | 1.82 |  |
| No |  | REF |  |  |  |  | REF |  |  |  |
| Cigarettes (V067) |  |  |  |  | *** |  |  |  |  | *** |
| Yes |  | 2.93 | 2.62 | 3.28 |  |  | 1.56 | 1.34 | 1.81 |  |
| No |  | REF |  |  |  |  | REF |  |  |  |
| Illicit drugs (V094) |  |  |  |  | *** |  |  |  |  | *** |
| Yes |  | 3.46 | 3.04 | 3.95 |  |  | 1.68 | 1.41 | 2.01 |  |
| No |  | REF |  |  |  |  | REF |  |  |  |
| **PAST MONTH** |  |  |  |  |  |  |  |  |  |  |
| Has any friend drunk in your presence? (V093) |  |  |  |  | *** |  |  |  |  | *** |
| Yes |  | 2.17 | 1.94 | 2.42 |  |  | 1.34 | 1.19 | 1.51 |  |
| No |  | REF |  |  |  |  | REF |  |  |  |
| Has any friend used illicit drugs in your presence? (V099) |  |  |  |  | *** |  |  |  |  | *** |
| Yes |  | 2.91 | 2.58 | 3.28 |  |  | 1.56 | 1.35 | 1.80 |  |
| No |  | REF |  |  |  |  | REF |  |  |  |

*Note:* Model adjusted for gender, age, school location (urban or rural) and type of school (public or private). REF = reference value. *** p<0.001.

**Supplementary Table 3**

Logistic regression models considering the adjusted and crude odds ratio regarding in emotional distress between adolescents categorized as of sexual violence (VSV, *n=* 20,492) and non-victims of sexual violence (nVSV, *n=*109,461). (Variable code: 160E).

| **VARIABLES CODED** |  | **Adjusted OR** | | | |  | **Crude OR** | | | |
| --- | --- | --- | --- | --- | --- | --- | --- | --- | --- | --- |
|  |  |  | **95% CI** | |  |  |  | **95% CI** | |  |
|  |  |  | **Lower** | **Upper** | ***p*** |  |  | **Lower** | **Upper** | ***p*** |
| **PAST MONTH** |  |  |  |  |  |  |  |  |  |  |
| **How often did your parent or guardian comprehend your concerns? (V102)** |  |  |  |  | ******* |  |  |  |  | ******* |
| Rarely |  | 2.89 | 2.58 | 3.23 |  |  | 1.98 | 1.75 | 2.24 |  |
| Sometimes |  | 2.00 | 1.76 | 2.26 |  |  | 1.68 | 1.48 | 1.91 |  |
| Most of the time |  | 1.45 | 1.28 | 1.65 |  |  | 1.36 | 1.20 | 1.55 |  |
| Always |  | REF |  |  |  |  | REF |  |  |  |
| **How often did you feel sad? (V112)** |  |  |  |  | *** |  |  |  |  | *** |
| Rarely |  | REF |  |  |  |  | REF |  |  |  |
| Sometimes |  | 1.63 | 1.47 | 1.82 |  |  | 1.34 | 1.20 | 1.51 |  |
| Most of the time |  | 2.60 | 2.32 | 2.90 |  |  | 1.51 | 1.31 | 1.73 |  |
| Always |  | 4.03 | 3.58 | 4.54 |  |  | 1.86 | 1.57 | 2.19 |  |
| **How often did you feel that no one cares about you? (V113)** |  |  |  |  | *** |  |  |  |  | 0.09 |
| Rarely |  | REF |  |  |  |  | REF |  |  |  |
| Sometimes |  | 1.47 | 1.33 | 1.62 |  |  | 1.06 | 0.96 | 1.18 |  |
| Most of the time |  | 2.19 | 1.98 | 2.43 |  |  | 1.17 | 1.04 | 1.33 |  |
| Always |  | 2.63 | 2.37 | 2.92 |  |  | 1.09 | 0.95 | 1.26 |  |
| **How often did you experience irritation, anger, or a negative mood? (V114)** |  |  |  |  | *** |  |  |  |  | *** |
| Rarely |  | REF |  |  |  |  | REF |  |  |  |
| Sometimes |  |  |  |  |  |  | 1.15 | 1.02 | 1.28 |  |
| Most of the time |  | 1.92 | 1.71 | 2.15 |  |  | 1.31 | 1.16 | 1.47 |  |
| Always |  | 2.59 | 2.30 | 2.91 |  |  | 1.42 | 1.24 | 1.62 |  |
| **How often did you sense life's lack of value? (V115)** |  | 1.33 | 1.19 | 1.48 | *** |  |  |  |  | *** |
| Rarely |  | REF |  |  |  |  | REF |  |  |  |
| Sometimes |  | 2.45 | 2.20 | 2.73 |  |  | 1.23 | 1.10 | 1.37 |  |
| Most of the time |  | 2.85 | 2.57 | 3.16 |  |  | 1.43 | 1.26 | 1.63 |  |
| Always |  | 1.74 | 1.57 | 1.92 |  |  | 1.42 | 1.23 | 1.64 |  |
| **How would you rate your health? (V175)** |  |  |  |  |  |  |  |  |  | *** |
| Very good |  | REF |  |  | *** |  | REF |  |  |  |
| Good |  | 1.12 | 1.02 | 1.24 |  |  | 0.97 | 0.88 | 1.07 |  |
| Fair |  | 1.69 | 1.53 | 1.86 |  |  | 1.16 | 1.04 | 1.29 |  |
| Bad |  | 2.87 | 2.46 | 3.36 |  |  | 1.66 | 1.41 | 1.97 |  |
| Very bad |  | 3.22 | 2.53 | 4.11 |  |  | 1.72 | 1.33 | 2.24 |  |

*Note:* Model adjusted for gender, age, school location (urban or rural) and type of school (public or private). REF = reference value. *** p<0.001.

**Supplementary Table 4**

Logistic regression models considering the adjusted and crude odds ratio regarding in emotional distress between adolescents categorized as rape victims (RV, n= 8,133) and non- rape victims (nRV, n= 121,820).

. (Variable code: 167E).

| **VARIABLES CODED** |  | **Adjusted OR** | | | |  | **Crude OR** | | | |
| --- | --- | --- | --- | --- | --- | --- | --- | --- | --- | --- |
|  |  |  | **95% CI** | |  |  |  | **95% CI** | |  |
|  |  |  | **Lower** | **Upper** | ***p*** |  |  | **Lower** | **Upper** | ***p*** |
| **PAST MONTH** |  |  |  |  |  |  |  |  |  |  |
| **How often did your parent or guardian comprehend your concerns? (V102)** |  |  |  |  | *** |  |  |  |  | *** |
| Rarely |  | 2.67 | 2.25 | 3.16 |  |  | 1.72 | 1.43 | 2.08 |  |
| Sometimes |  | 1.75 | 1.44 | 2.12 |  |  | 1.53 | 1.25 | 1.86 |  |
| Most of the time |  | 1.11 | 0.90 | 1.36 |  |  | 1.09 | 0.89 | 1.34 |  |
| Always |  | REF |  |  |  |  | REF |  |  |  |
| **How often did you feel sad? (V112)** |  |  |  |  | *** |  |  |  |  | * |
| Rarely |  | REF |  |  |  |  | REF |  |  |  |
| Sometimes |  | 1.32 | 1.13 | 1.56 |  |  | 1.15 | 0.97 | 1.37 |  |
| Most of the time |  | 2.56 | 2.17 | 3.01 |  |  | 1.43 | 1.16 | 1.77 |  |
| Always |  | 4.30 | 3.64 | 5.07 |  |  | 1.65 | 1.29 | 2.10 |  |
| **How often did you feel that no one cares about you? (V113)** |  |  |  |  | *** |  |  |  |  | 0.15 |
| Rarely |  | REF |  |  |  |  | REF |  |  |  |
| Sometimes |  | 1.45 | 1.24 | 1.68 |  |  | 1.11 | 0.94 | 1.30 |  |
| Most of the time |  | 2.47 | 2.13 | 2.87 |  |  | 1.31 | 1.08 | 1.59 |  |
| Always |  | 3.28 | 2.83 | 3.80 |  |  | 1.22 | 0.98 | 1.51 |  |
| **How often did you experience irritation, anger, or a negative mood? (V114)** |  |  |  |  | *** |  |  |  |  | *** |
| Rarely |  | REF |  |  |  |  | REF |  |  |  |
| Sometimes |  | 0.97 | 0.82 | 1.13 |  |  | 0.84 | 0.72 | 0.99 |  |
| Most of the time |  | 1.49 | 1.27 | 1.74 |  |  | 0.96 | 0.81 | 1.14 |  |
| Always |  | 2.00 | 1.71 | 2.35 |  |  | 0.91 | 0.76 | 1.09 |  |
| **How often did you sense life's lack of value? (V115)** |  |  |  |  | *** |  |  |  |  | *** |
| Rarely |  | REF |  |  |  |  | REF |  |  |  |
| Sometimes |  | 1.77 | 1.52 | 2.06 |  |  | 1.32 | 1.12 | 1.57 |  |
| Most of the time |  | 2.99 | 2.56 | 3.50 |  |  | 1.84 | 1.52 | 2.22 |  |
| Always |  | 4.19 | 3.64 | 4.83 |  |  | 2.21 | 1.81 | 2.70 |  |
| **How would you rate your health? (V175)** |  |  |  | *** |  |  |  |  |  | *** |
| Very good |  | REF |  |  |  |  | REF |  |  |  |
| Good |  | 0.92 | 0.79 | 1.06 |  |  | 0.82 | 0.70 | 0.95 |  |
| Fair |  | 1.43 | 1.23 | 1.65 |  |  | 0.96 | 0.83 | 1.13 |  |
| Bad |  | 2.80 | 2.27 | 3.46 |  |  | 1.51 | 1.20 | 1.90 |  |
| Very bad |  | 5.02 | 3.80 | 6.64 |  |  | 2.45 | 1.82 | 3.30 |  |

*Note:* Model adjusted for gender, age, school location (urban or rural) and type of school (public or private). REF = reference value. * *p<* 0.05; *** *p<*0.001.
